# Genome-Wide Analysis of the *TCP* Gene Family and Their Expression Pattern Analysis in Tea Plant (*Camellia sinensis*)

**DOI:** 10.3389/fpls.2022.840350

**Published:** 2022-07-01

**Authors:** Xiaowen Shang, Zhaolan Han, Dayan Zhang, Ya Wang, Hao Qin, Zhongwei Zou, Lin Zhou, Xujun Zhu, Wanping Fang, Yuanchun Ma

**Affiliations:** ^1^College of Horticulture, Nanjing Agricultural University, Nanjing, China; ^2^School of Life Sciences, Southern University of Science and Technology, Shenzhen, China; ^3^Agricultural and Forestry Service Center, Suzhou, China; ^4^Department of Plant Science, University of Manitoba, Winnipeg, MB, Canada; ^5^Forestry and Pomology Research Institute, Shanghai Academy of Agricultural Sciences, Shanghai, China

**Keywords:** genome-wide analysis, *TCP* gene family, evolution, expression pattern, *Camellia sinensis*

## Abstract

TEOSINTE BRANCHED1/CYCLOIDEA/PCF (TCP) transcription factors TEOSINTE BRANCHED1/CYCLOIDEA/PCF have been suggested to control the cell growth and proliferation in meristems and lateral organs. A total of 37 *CsTCP* genes were identified and divided into two classes, class I (PCF, group 1) and class II (CIN CYC/TB1, groups 2, and 3). The residues of TEOSINTE BRANCHED1/CYCLOIDEA/PCF of Camellia sinensis (Tea plant) (CsTCP) proteins between class I and class II were definitely different in the loop, helix I, and helix II regions; however, eighteen conserved tandem was found in bHLH. There are a large number of *CsTCP* homologous gene pairs in three groups. Additionally, most CsTCP proteins have obvious differences in motif composition. The results illuminated that CsTCP proteins in different groups are supposed to have complementary functions, whereas those in the same class seem to display function redundancies. There is no relationship between the number of *CsTCP* gene members and genome size, and the *CsTCP* gene family has only expanded since the divergence of monocots and eudicots. WGD/segmental duplication played a vital role in the expansion of the *CsTCP* gene family in tea plant, and the *CsTCP* gene family has expanded a lot. Most *CsTCP* genes of group 1 are more widely and non-specifically expressed, and the *CsTCP* genes of group 2 are mainly expressed in buds, flowers, and leaves. Most genes of group 1 and some genes of group 2 were up-/downregulated in varying degrees under different stress, *CsTCP* genes of group 3 basically do not respond to stress. *TCP* genes involved in abiotic stress response mostly belong to PCF group. Some *CsTCP* genes may have the same function as the homologous genes in Arabidopsis, but there is functional differentiation.

## Introduction

As an important economical crop, the tea plant (*Camellia sinensis*) is widely planted in more than 52 countries across the world (Li et al., 2017b). The tea leaves are the main source of the most popular natural non-alcoholic beverages (Chen et al., 2007; Zhang et al., 2015). In tea plant, shoot branching greatly affects the overall plant architecture and other traits of plant, such as height, light harvesting efficiency, and leaf production, which influences the costs and benefits of agricultural production.

Branches are generated from axillary meristems of the axils of leaves, and then, the patterns of branching are conserved in angiosperms. There are few reports about branch development that plays a key role in the life of tea plant. Recently, Cao et al. (2020) revealed that zigzag-shaped shoot formation might be associated with the gravitropism response and polar auxin transport in tea plants (Cao et al., 2020). In addition, Yu et al. (2021) showed that the profiling of more than 40 developmental-related genes (*CYC/GROWTH REGULATING FACTORs* (*GRFs*), *COTYLEDON 1 FACTORs* (*GIFs*), *CUP-SHAPED*, *PHAVOLUTA* (*PHV*), and *REVOLUTA* (*REV*)) essentially proved their high expression levels in developing tea plant buds and leaves (Yu et al., 2021). However, the transcription factors that regulate the growth and development of shoot tips and the formation of tissues and organs in tea plant are rarely studied.

TEOSINTE BRANCHED1/CYCLOIDEA/PCF (TCP) transcription factors (TEOSINTE BRANCHED1/CYCLOIDEA/PCF) have been suggested to control the cell growth and proliferation in meristems and lateral organs (Martin-Trillo and Cubas, 2010). TCP domain was initially identified in four proteins encoded unrelated genes, from which the name “TCP” was derived: *Teosinte branched1 (TB1)* from maize (*Zea mays*), which participates in regulating apical dominance, inflorescence development, and some other processes of broad interest in maize developmental biology (Doebley et al., 1997); *CYCLOIDEA (CYC)* from snapdragon (*Antirrhinum majus*) (Luo et al., 1996) which regulates floral asymmetry, and the *PROLIFERATING CELL FACTORS 1* and *2* (*PCF1* and *PCF2*) from rice (*Oryza sativa*) which is involved in cell growth and proliferation in meristems and lateral organs (Kosugi and Ohashi, 1997).

*TCP* gene family is a transcription factor (TF), which contained a conserved non-canonical basic-helix-loop-helix (bHLH) domain with 59 conserved amino acid residues (Cubas et al., 1999). As plant-specific transcription factors, *TCP* genes are identified in basal land plant and freshwater algal genomes, such as in the *Arabidopsis thaliana*, poplar, rice, club-moss, and moss genomes, even in *Rhodophyta* and *Prasinophyceae* (Navaud et al., 2007). TCP proteins have been divided into two classes (Cubas, 2002), class I (PCF) and class II (CIN and CYC/TB1). Based on the widely existence of *TCP* genes in plants, TCP proteins are required for the multiple developmental pathways, especially in plant morphologies. Previous studies have reported their involvements in shoot branching (Martín-Trillo et al., 2011), controlling apical dominance (Doebley et al., 1997), and the formation of meristematic tissue (Faivre-Rampant et al., 2004). In addition, they also participated in the leaf and floral development (Luo et al., 1996; Palatnik et al., 2003; Aguilar-Martínez and Sinha, 2013), senescence (Huang and Irish, 2015), flavonoid biosynthesis (Li and Zachgo, 2013), plant immunity (Schommer et al., 2008; Lopez et al., 2015), and hormonal signaling including jasmonic acid (Schommer et al., 2008; Danisman et al., 2012), gibberellin (Daviere et al., 2014), and auxin (Li and Zachgo, 2013).

A total of 24 TCP proteins in *Arabidopsis* were divided into two classes. Class I (TCP-P, also known as PCF) have been indicated to function as the positive regulators of cell proliferation (Kosugi and Ohashi, 2002). For example, AtTCP14, AtTCP15 (Kieffer et al., 2011), AtTCP20 (Li et al., 2005; HERVé et al., 2009), AtTCP7, AtTCP8, AtTCP22, and AtTCP23 (Aguilar-Martínez and Sinha, 2013) proteins have been reported to play the important roles in processes of cell division and proliferation in leaf growth and development. Class II (TCP-C, also known as CIN and CYC/TB1), which duplicate from ancestral *tb1*-like gene, have been indicated to function as the regulators of branching signals within axillary buds and the morphogenesis of shoot lateral organs (Aguilar-MARTíNEZ et al., 2007; Finlayson, 2007; Koyama et al., 2007; Mitsuda et al., 2010). Thus, they will induce the plant branching and meristematic activity (Aguilar-MARTíNEZ et al., 2007; Koyama et al., 2007; Mitsuda et al., 2010). For instance, ectopic expression of *AtTCP3* inhibits the formation of shoot meristem (Koyama et al., 2007). *AtTCP5* is considered as an enhancer in axillary branch outgrowth (van Es et al., 2019), and *AtTCP12* (*BRANCHED2*) and *AtTCP18* (*BRANCHED1*) were involved in branching control (Aguilar-MARTíNEZ et al., 2007). Obviously, functional redundancy has been inferred in various members of the TCP groups (Cubas et al., 1999; Danisman et al., 2013), such as *AtTCP14* and *AtTCP15* (Kieffer et al., 2011; Steiner et al., 2012; van Es et al., 2019), which affect internode length and leaf shape and induce the branching and meristematic activity.

In *Solanum lycopersicum*, several genes play a key role in ripening, such as *SlTCP12*, *SlTCP15*, and *SlTCP18* (Parapunova et al., 2014). In tobacco (*Nicotiana tabacum*), several *TCP* genes can affect the leaf development and growth such as *NtTCP18* (Chen et al., 2016). There are 22 *OsTCP* genes in rice. The OsTCP proteins were divided into three groups, PCF, CIN, and CYC/TB1 groups (Yao et al., 2007). Overexpressing *OsTB1* transgenic rice exhibited significant reduced lateral branch without the propagation of axillary buds being affected, which indicates that *OsTB1* gene negatively regulates lateral branchings (Takeda et al., 2003). A total of 38 *TCP* genes were identified in *Gossypium raimondii* (Ma et al., 2014). Among them, the RNAi silenced *GbTCP* (GenBank accession no. DQ912941) transgenic line produced shorter fiber, a reduced lint percentage, and a lower fiber quality than the wild-type plants and overexpression of *GbTCP* in *Arabidopsis* enhanced root hair initiation and elongation. It obviously indicated that *GbTCP* regulated the fiber elongation and root hair development (Hao et al., 2012; Wang et al., 2013). A total of 52 *TCP* genes were identified in apple (*Malus domestica*) genome which were divided into three classes (classes 1, 2, and 3) (Xu et al., 2014).

In this study, 37 TCP proteins were identified in *Camellia sinensis*. The structural features, phylogenetic relations, and interaction networks of TEOSINTE BRANCHED1/CYCLOIDEA/PCF of Camellia sinensis (Tea plant) (CsTCP) proteins were analyzed. The expression profiles of 37 *CsTCP* genes in eight different tissues were surveyed to investigate their biological functions.

## Materials and Methods

### Genome-Wide Identification of *TCP* Genes in Tea Plant

The AtTCP protein sequence file was downloaded from the Arabidopsis Information Resource (TAIR)^1^ and put the AtTCP protein sequence on the Pfam protein analysis professional website^2^ search to obtain the hidden Markov model (HMM) profile of TCP domain (PF03634) (Eddy, 1998). The program HMMER 3.0 was used to search for CsTCP protein members in the tea plant protein sequence file (*E*-value < 1.0) that was downloaded from the Tea Plant Information Archive (TPIA)^3^ (Xia et al., 2019), and then, we acquired the protein sequence of *CsTCP* candidate genes. The conserved domains of candidate TCP proteins were identified one by one using the online websites of Pfam and SMART,^4^ and some sequences that did not contain TCP domains were removed.

### Analyses of Phylogenetic Tree

The amino acid sequences of TCP proteins from *Vitis vinifera*, *Arabidopsis thaliana*, and *Zea mays* were obtained from the Plant Transcription Factor Databases.^5^ The amino acid sequences of all TCP proteins of *Oryza sativa* were derived from Rice Genome Annotation Project.^6^ The TCP proteins of *Antirrh-inum majus* were obtained from snapdragon genome database.^7^ All the TCP proteins in this study were aligned using MAFFT 7.0 (Katoh and Standley, 2013). A phylogenetic tree was constructed using the maximum likelihood estimate (ML) method by RAxML 8.0 software (Stamatakis, 2014).

### Characteristics of *TCP* Proteins Analysis

The primary structure of TCP proteins was predicted using ProtParam tool.^8^ The Softberry Web Site^9^ was used to predict the subcellular localization of TCP proteins. The MEME (E < 1e-10) (Bailey et al., 2009)^10^ program was used to analyze protein structural motifs and set the maximum number of output motifs to 10. The DNAMAN 7 (Lynnon Corporation) was used to align the CsTCP domain sequences.

### Gene Sequence Analysis for *CsTCPs*

Exon–intron structures of *CsTCP* genes were identified and visualized using TBtools (Chen et al., 2020a). *Cis*-element analysis of the 2,000 bp upstream sequences of each *CsTCP* gene at the five end of the cDNA was predicted using Plantcare program.^11^ Tandem duplications of *TCP* genes in the tea genome were identified by checking physical locations within a 200-kb adjacent region in individual chromosomes. The information for homologous gene pairs and syntenic relationships between tea plant and other species was analyzed using MCscan and using TBtools for visualization^12^ (Wang et al., 2012).

### Mapping *CsTCP* on Chromosomes

*CsTCP* genes were mapped on chromosomes based on the whole-genome annotation from TPIA. The map was generated in the MapInspect software.

### Plant Materials

About 1-year-old tea plant cultivars (*C. sinensis* cv. “longjing43”) were planted in an illuminating incubator at the Tea Science Research Institute, College of Horticulture, Nanjing Agriculture University, Jiangsu Province, China (32°03′ N, 118°46′E). The set of the incubator was controlled at 22°C temperature, 14/10 h (day/night).

### Expression Pattern Analysis

About 2 weeks after raising seedlings in illuminating incubator, different development stages of tea leaves were sampled including a bud with first leaf (I), 2nd and 3rd leaves (II), natural leaves (4th, 5th, 6th leaves, III), and roots (IV), the epidermis and vascular tissue of unlignified stem (tender phloem (V), tender xylem (VI)) and lignified stem 9older phloem (VII), older xylem (VIII)] (Supplementary Figure 1). All the samples were frozen in liquid nitrogen and stored at –80°C for the following steps. The RNA of samples was extracted using RNA prep Pure Plant Kit (Polysaccharides and Polyphenolics-rich) from TIANGEN (Tiangen Biotech Co., Ltd., Beijing, China). The first-strand cDNA was synthesized using HiScript^®^ II Q RT Super Mix (TaKaRa Biotech Co., Ltd., Dalian, China). The quantitative PCR primers were designed by Beacon designer 7.0 (Supplementary Table 1). Quantitative PCR was conducted in Switzerland Roche, Light Cycler^®^ 480 II using SYBR GREEN dye (TaKaRa Biotech Co., Ltd., Dalian, China). The thermos cycle was set as follows: 95°C for 30 s; 40 cycles of 95°C for 10 s, and 60°C for 30 s. β*-actin*, as a reference gene of *Camellia sinensis*, was used as an internal control (Li et al., 2017a). Quantitative expression analysis in each sample was carried out with each of three biological and technique replicates. Relative gene expressions were analyzed using the 2-ΔΔCt method (Livak and Schmittgen, 2001).

### Statistical Analysis

The experimental data were sorted and statistically analyzed using Excel 2019 (Microsoft Corp, Albuquerque, United States) software, significance analysis was performed using IBM SPSS Statistics 20.0 (IBM Corporation, New York, United States), all data analysis results were expressed as mean (*n* = 3) ± standard deviation (SD), and different lowercase letters indicate significant difference at *p* < 0.05 level. Graphs were made using GraphPad 8.0.1 (GraphPad Software, San Diego, United States) and TBtools v1.098.

## Results

### Identification and Sequence Analysis of *TCP* Gene Family in Tea Plant

A total of 39 candidate Cs*TCP* genes were obtained from the “Shuchazao” genome database TPIA (SCZ), and two of them were excluded: *TEA005756.1* and *TEA027571.1* due to the lack of TCP domain. Finally, 37 genes were renamed and included in this study (Table 1). The genome database of *Camellia sinensis* var. sinensis (CSS) cv. Huangdan (HD)(Wang et al., 2021) and Tieguanyin (TGY)(Zhang et al., 2021) with good assembly quality is worthy of reference. Therefore, we used the same method to identify the *CsTCP* gene family in the varieties of tea plant HD and TGY and constructed a phylogenetic tree to correspond to the CsTCP proteins. The maximum number of TCP proteins retrieved in SCZ was 37, 32 in HD, and 36 in TGY, all of which contained TCP-conserved domains (Supplementary Table 1). The annotation information of “Shuchazao” genome database is relatively perfect and widely used, and the number of TCP identified is the largest. Therefore, we analyze and discuss the TCP protein identified in SCZ.

**TABLE 1:** Analysis of amino acid sequence characteristics of *CsTCP* gene family in tea plants.

Gene name	Gene ID	Number of amino acids	Molecular weight	Theoretical pI	GRAVY	Subcellular localization
CsTCP1	TEA018731.1	422	47620.50	9.34	–0.802	Extracellular
CsTCP2	TEA014952.1	516	55893.43	10.52	–0.606	Nuclear
CsTCP3	TEA011977.1	350	39302.74	7.23	–0.865	Extracellular
CsTCP4	TEA021545.1	392	43354.66	6.57	–0.617	Extracellular
CsTCP5	TEA019201.1	419	44501.85	6.85	–0.723	Extracellular
CsTCP6	TEA012645.1	362	40361.98	9.32	–0.576	Extracellular
CsTCP7	TEA028581.1	351	37802.02	9.11	–0.604	Nuclear
CsTCP8	TEA013742.1	369	39090.35	6.93	–0.549	Nuclear
CsTCP9	TEA003322.1	521	55340.00	6.81	–0.689	Nuclear
CsTCP10	TEA033771.1	423	45234.56	6.70	–0.678	Nuclear
CsTCP11	TEA003956.1	317	33325.22	9.54	–0.428	Nuclear
CsTCP12	TEA027566.1	172	19842.51	10.00	–0.950	Nuclear
CsTCP13	TEA012894.1	254	26446.76	9.71	–0.278	Nuclear
CsTCP14	TEA013055.1	252	27452.53	8.99	–0.719	Nuclear
CsTCP15	TEA021348.1	531	59443.96	6.29	–0.946	Nuclear
CsTCP16	TEA032594.1	311	33329.09	8.64	–0.728	Nuclear
CsTCP17	TEA027172.1	333	34886.93	8.67	–0.545	Nuclear
CsTCP18	TEA015531.1	275	29601.43	9.47	–0.453	Nuclear
CsTCP19	TEA015978.1	395	43068.95	5.87	–0.772	Nuclear
CsTCP20	TEA024520.1	351	36671.57	6.78	–0.238	Nuclear
CsTCP21	TEA014746.1	368	39212.91	5.67	–0.443	Nuclear
CsTCP22	TEA015233.1	437	49169.43	6.77	–0.482	Extracellular
CsTCP23	TEA007156.1	380	40088.31	8.96	–0.365	Nuclear
CsTCP24	TEA014573.1	349	39268.48	6.76	–0.900	Extracellular
CsTCP25	TEA000693.1	351	36810.12	8.78	–0.095	Nuclear
CsTCP26	TEA018080.1	544	60323.46	6.04	–0.844	Extracellular
CsTCP27	TEA009154.1	336	37332.29	6.41	–0.753	Nuclear
CsTCP28	TEA005508.1	392	43920.99	8.96	–0.735	Extracellular
CsTCP29	TEA030615.1	392	43889.05	9.11	–0.722	Extracellular
CsTCP30	TEA021025.1	434	47546.11	9.76	–0.280	Extracellular
CsTCP31	TEA017971.1	346	36780.06	9.82	–0.365	Extracellular
CsTCP32	TEA032820.1	150	16821.12	8.74	–0.381	Nuclear
CsTCP33	TEA021647.1	216	23674.41	5.10	–0.497	Extracellular
CsTCP34	TEA006139.1	607	67587.59	6.05	–0.263	Nuclear
CsTCP35	TEA025851.1	254	27307.48	6.21	–0.538	Nuclear
CsTCP36	TEA033591.1	206	22319.29	7.82	–0.344	Nucleus
CsTCP37	TEA021816.1	363	40548.19	9.39	–0.588	Nuclear

**FIGURE 1:**
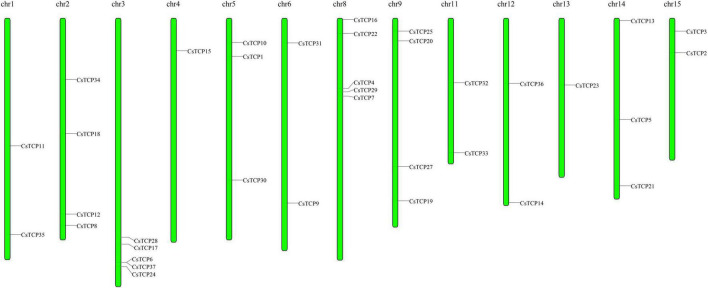
The chromosomal location of *CsTCP* genes.

The length of amino acids in 37 *CsTCP* genes is ranged from 150 (*CsTCP32*) to 607 (*CsTCP34*). The pI of 56% of the CsTCP proteins was more than 7. In addition, the molecular weights of 37 CsTCP proteins are ranged from 16.8 kDa (CsTCP32) to 67.6 kDa (CsTCP34) (Table 1). A total of thirty-six *CsTCP* genes were mapped to 13 chromosomes (Chr) (Figure 1), and *CsTCP26* was the only one that is not assembled on the chromosome. The distribution of *CsTCP* genes was uneven across all of the chromosomes from Chr 1–Chr 6, Chr 8, Chr 9, and Chr 11–Chr 15. Most *TCP* genes were found on Chr 3 (*CsTCP6*, *CsTCP17*, *CsTCP24*, *CsTCP28*, and *CsTCP37*) and Chr 8 (*CsTCP4*, *CsTCP7*, *CsTCP16*, *CsTCP22*, and *CsTCP29*). Chr 4 and Chr 13 had only one *CsTCP* gene (*CsTCP5* and *CsTCP23*).

### *CsTCP* Protein Sequence Analysis

**FIGURE 2:**
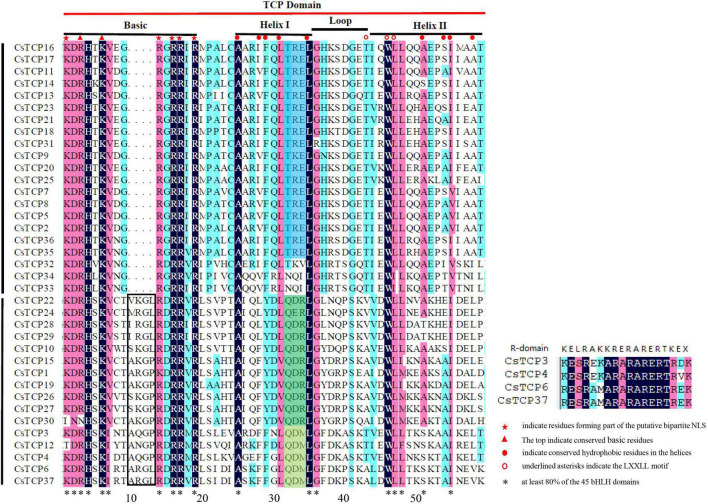
Multiple sequence alignment and protein sequence signs of the TCP domain.

To clarify the sequence characteristics of CsTCP proteins, we performed multiple sequence alignment. In TCP domain with 59 residues, basic region is the most conservative, helix is less conservative, and loop region changes greatly (Yao et al., 2007). The basic-helix-loop-helix (bHLH) domain of CsTCP proteins is very similar to that of rice, Arabidopsis, and grape, indicating that the TCP domain is highly conserved among different species. We identified 18 residues that were identical in at least 80% of the 45 bHLH domains (Figure 2): 10 in the basic region (KDRHXKVXXRRRX R), seven hydrophobic residues in the two helices (21-A, 27-L, 31-L, 42-W, 43-L, 44-L, 51-I in our alignment), and a helix-breaking glycine (32-G in our alignment) in the loop between the helices (Figure 2). In addition to the bHLH domain, four CsTCP proteins shared an R domain comprising conserved polar residues (Figure 2).

Based on the characteristics of TCP domain, CsTCP proteins are divided into two class: class I and class II. Class I is PCF group (group 1) which contains 21 CsTCP proteins; class II contains two group, CIN and CYC/TB1. CIN group (group 2) has 11 CsTCP proteins and CYC/TB1 group (group 3) has 5 CsTCP proteins. Each group has unique sequence and structural characteristics. For example, in the basic region, PCF group has four amino acid residues less than CIN and CYC/TB1 group, which makes that the two types of TCP proteins have different but similar DNA-binding sites (class I is GGNCCAC and class II is GTGGNCCC). In terms of helix I and loop region residues, members of each group have obvious and unique sequence characteristics. For example, of the last three residues in the helix I region, members of group 1 were TRE, group 2 members were QDR, and group 3 members were QDM (Figure 2).

**FIGURE 3:**
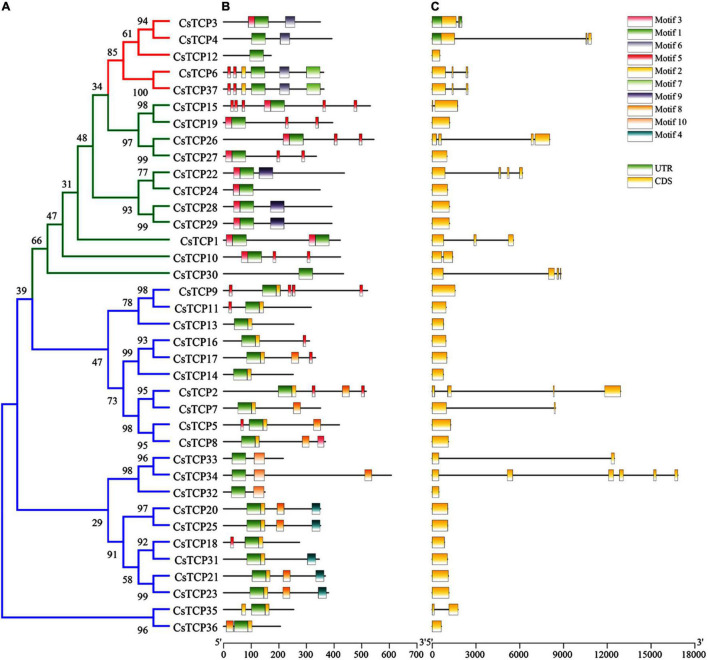
CsTCP protein and gene sequences analysis. **(A)** Phylogenetic tree (ML), the blue line represents the PCF group, the green line represents the CIN group, and the red line represents the CYC/TB1 group; **(B)** motif analysis; **(C)** gene structure analysis, horizontal lines indicate introns.

A total of thirty-seven CsTCP proteins were constructed into a phylogenetic tree (Figure 3A), and it was found that there were 15 pairs of homologous genes in the three groups, 9 pairs in the PCF group, *CsTCP9*/*CsTCP11*, *CsTCP16*/*CsTCP17*, *CsTCP2*/*CsTCP7*, *CsTCP5*/*CsTCP8*, *CsTCP33*/*CsTCP34*, *CsTC P20*/*CsTCP25*, *CsTCP18*/*CsTCP31*, *CsTCP21/CsTCP23*, and *CsTCP35*/*CsTCP36*; CIN group 4 pairs, *CsTCP15*/*CsTCP19*, *CsTCP22*/*CsTCP24*, *CsTCP26*/*CsTCP27*, *CsTCP28*/*CsTCP29*; *CsTCP3*/*CsTCP4*, and *CsTCP6*/*CsTCP37*. The homologous gene pairs showed similarities in gene structure and the motif composition of translated proteins. Among them, four pairs of homologous genes *CsTCP20*/*CsTCP25*, *CsTCP21*/*CsTCP23*, *CsTCP28*/*CsTCP29*, and *CsTCP6*/*CsTCP37* had exactly the same gene structure and the motif compositions of the post-translational proteins (Figures 3B,C).

There are also large differences in the motif composition between the two class. A total of ten regular motifs were identified in CsTCP proteins (Figure 3B and Supplementary Figure 2). Motif 1 was found to distribute in TCP domain regions, so it exists in all CsTCP proteins. Motif 6 was distributed in R-domain regions. Most members of group 3 had an R domain (Figure 1), suggesting that they may have additional activity (Cubas et al., 1999). The proteins of PCF group mostly contain motif 2, and only CsTCP32, CsTCP33, and CsTCP34 do not contain motif 2, but they contain motif 10 which is not present in any other CsTCP proteins. A total of 11 CsTCP proteins contain motif 8 which is only present in the proteins of PCF groups. Except CsTCP30, all proteins of CIN group contain motif 3; the proteins of CYC/TB1 group contain motif 6, except CsTCP12. In addition, there are some interesting phenomena. For example, motif 5 exists in 14 TCP proteins, but most CsTCP proteins in class I contain only one motif 5, whereas CsTCP proteins in class II basically contain multiple motif 5. Some unique motifs exist only in certain CsTCPs, such as motif 4 exists only in CsTCP20/CsTCP25/CsTCP21/CsTCP23/CsTCP31, motif 9 exists only in CsTCP22/CsTCP28/CsTCP29, and motif 7 exists only in CsTCP6/CsTCP37.

The *CsTCP* gene structures show that the *CsTCPs* introns number is between 0 and 3, except for *CsTCP34* which has 5 introns, and most of the *CsTCPs* (22/37) in tea plant have no intron (Figure 3C). Moreover, only *CsTCP3* and *CsTCP4* contain two and one UTR, respectively.

A total of five *cis*-acting elements were detected, which are participated in hormone response (229), stress response (53), and light response (405) and involved in growth and development regulation (28) and metabolism regulation (20) (Supplementary Figure 3). Among them, the proportion of light-responsive elements (55%) is the highest, followed by hormone-responsive elements (31%), stress-responsive elements (7%) and developmental and metabolic response elements (7%) are close, which is similar to PCF and CIN group, and CYC/TB1 group hormones (40%) accounted for more than other. Among hormone response elements, the ratio of abscisic acid (37%) and methyl jasmonate (33%) response elements was the highest, followed by auxin, gibberellin, and salicylic acid response elements.

### Evolutionary Analysis of the *CsTCP* Gene Family

The study explored the evolution of the *TCP* gene family in tea plants by constructing phylogenetic trees among different species, comparing genomic information of *TCP* genes, and performing collinear analysis.

**FIGURE 4:**
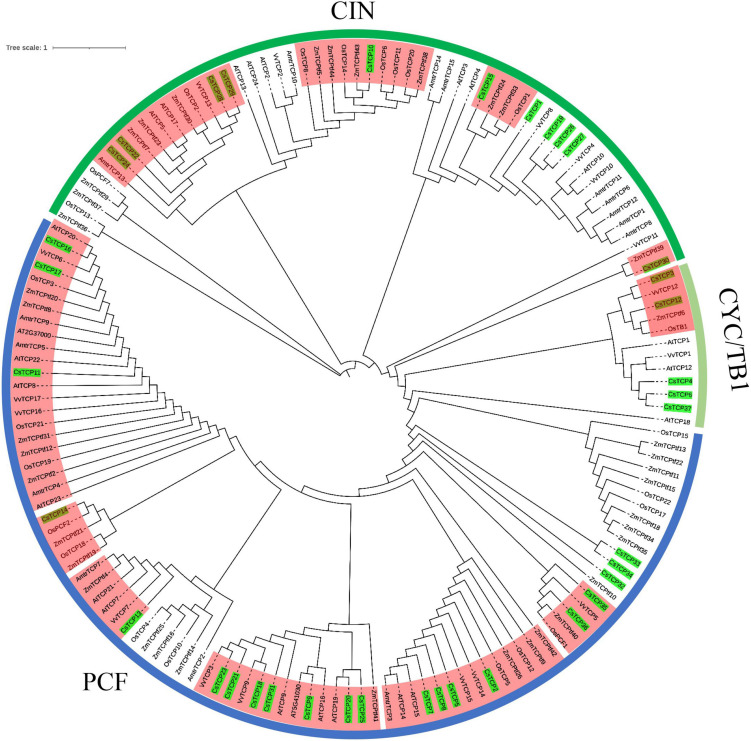
The phylogenetic analysis of *TCP* gene family among *Camellia sinensis, Arabidopsis thaliana*, *Oryza sativa, Zea mays, Vitis vinifera*, and *Antirrhinum majus*. The red shaded part is the clades containing both monocots and eudicots TCP proteins.

To study the evolution of *CsTCP* gene family, the phylogenetic tree was constructed by the TCP protein of *Camellia sinensis*, *Zea mays*, *Oryza sativa*, and *Antirrhinum majus* that three species first identified *TCP* genes, *Arabidopsis thaliana* that is the herbal model plant, and *Vitis vinifera* that is woody plant. All TCP proteins are divided into three groups: PCF, CIN, and CYC/TB1. Among them, there are 11 clades of TCP protein in monocots (rice and maize) and eudicots (snapdragon, Arabidopsis, grapevine, and tea plant) (Figure 4). In 4 clades, there are only TCP protein in tea and monocots. The TCP proteins of tea plant in 4 clades are CsTCP10, CsTCP14, CsTCP15, and CsTCP30, respectively. They may be new CsTCP proteins produced by the evolution in tea plant. In the phylogenetic tree, CsTCP proteins clustered together, mostly with TCP proteins of Arabidopsis and grapevine.

The number and proportion of *TCP* gene family in the nine species were compared and analyzed (Table 2). The number of *TCP* genes increases with the evolution of species from lower to higher. There are fewer *TCP* members in *P*. *patens* and *S*. *moellendorffii* than higher plants, which indicates that the *TCP* gene family has been expanded in higher plant. It is worth noting that the number of *TCP* genes in *P*. *patens* and *S*. *moellendorffii* is similar, and which in grapevine and snapdragon is also similar. But the genome size of moss is two times that of selaginella, the genome size of grape and snapdragon is similar. The number of *CsTCP* genes in tea is 1.5 times that of Arabidopsis, but the genome size of tea plant is 22 times that of Arabidopsis. It is found that the proportion of *CsTCP* gene members in the whole-genome in each species is not related to the genome size of species.

**TABLE 2:** The *TCP* transcription factors in genomes of nine species.

		Size of	Number of
Species	Total genes	genome (Mb)	*TCP*
*Physcomitrella patens*	35,938 (0.019%)	454	7
*Selaginella moellendorffii*	22,285 (0.026%)	212.5	6
*Oryza sativa*	49,061 (0.047%)	372	23
*Antirrhinum majus*	37,714 (0.053%)	520	20
*Amborella trichopoda*	26,846 (0.056%)	706	15
*Vitis vinifera*	26,346 (0.065%)	487	17
*Arabidopsis thaliana*	33,602 (0.071%)	135	24
*Zea mays*	38,620 (0.098%)	2,183	38
*Camellia sinensis*	33,932 (0.110%)	3,051	37

**FIGURE 5:**
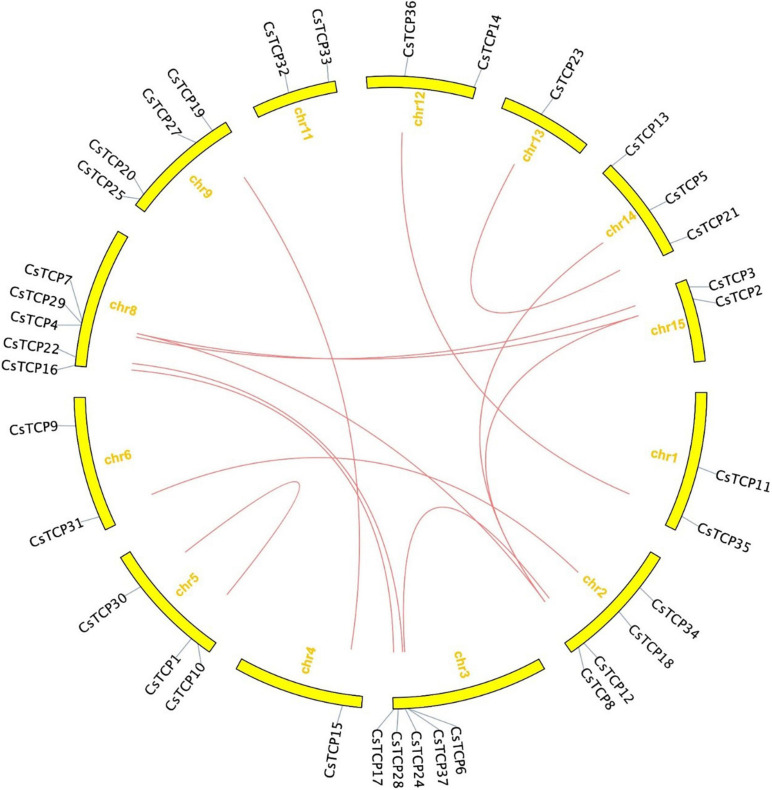
Syntenic genes among *TCP* gene family from *Camellia sinensis.*

There are 13 syntenic pairs in tea plant, nine of which are homologous gene pairs, *CsTCP2/CsTCP7*, *CsTCP5/CsTCP8*, *CsTCP16/CsTCP17*, *CsTCP18/CsTCP31*, *CsTCP21/CsTCP23*, *CsTCP35/CsTCP36*, *CsTCP15/CsTCP19*, *CsTCP22/CsTCP24*, *and CsTCP3/CsTCP4*. Moreover, there are four syntenic pairs that are non-homologous gene pairs, *CsTCP2/CsTCP8*, *CsTCP12/CsTCP37*, and *CsTCP1*/*CsTCP30* (Figure 5). Gene duplicated event analysis showed that the coordinates of transposed duplication and whole-genome duplication (WGD) events were detected in 35 *CsTCP* genes (Supplementary Table 2). *CsTCP26* and *CsTCP37* were not detected duplication events. The reason of former may be that it is not assembled on the chromosome.

**FIGURE 6:**
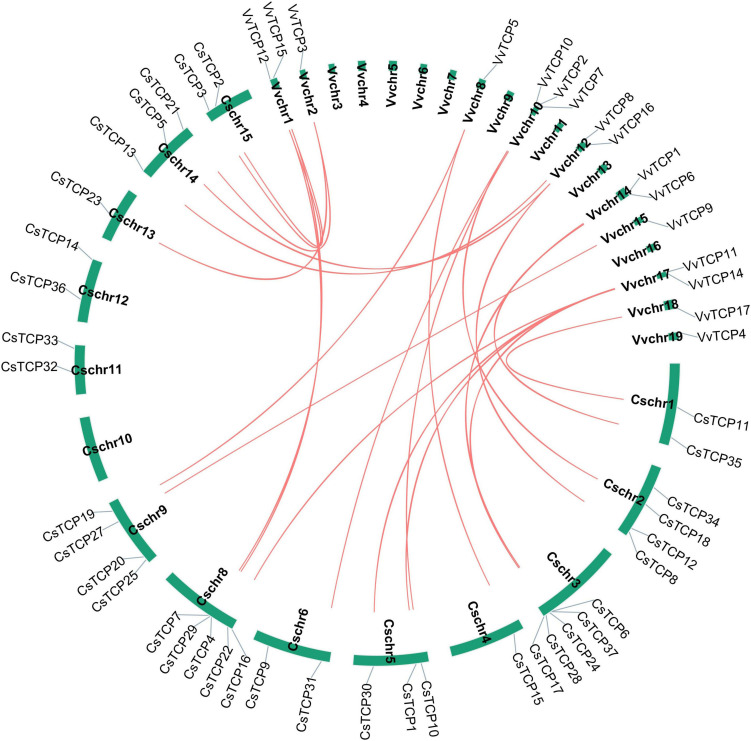
Syntenic genes among *TCP* gene family between *Camellia sinensis* and *Vitis vinifera.*

To further explore the origin and probable evolutionary mechanisms of the *CsTCP* gene family, we also investigated the syntenic blocks in tea plant and grapevine. A total of thirteen syntenic pairs were detected in tea plant, and 16 syntenic pairs were detected between tea plant and grapevine (Figure 6). The results showed that 16 *VvTCP* genes have syntenic counterpart in tea plants and *VvTCP13* was excluded. Interestingly, the syntenic counterparts in tea plant of *VvTCP*2/*VvTCP*4/*VvTCP*8/*VvTCP*10 of CIN group belong to PCF group (except *CsTCP10*). In CYC/TB1 group, *VvTCP1/CsTCP6*, *VvTCP1/CsTCP37*, *VvTCP11/CsTCP3*, and *VvTCP11/CsTCP4* are the syntenic pairs. *CsTCP3*, *CsTCP4*, *CsTCP6*, and *CsTCP37* belong to CYC/TB1 group.

### Expression Pattern Analysis of *CsTCP* Genes

**FIGURE 7:**
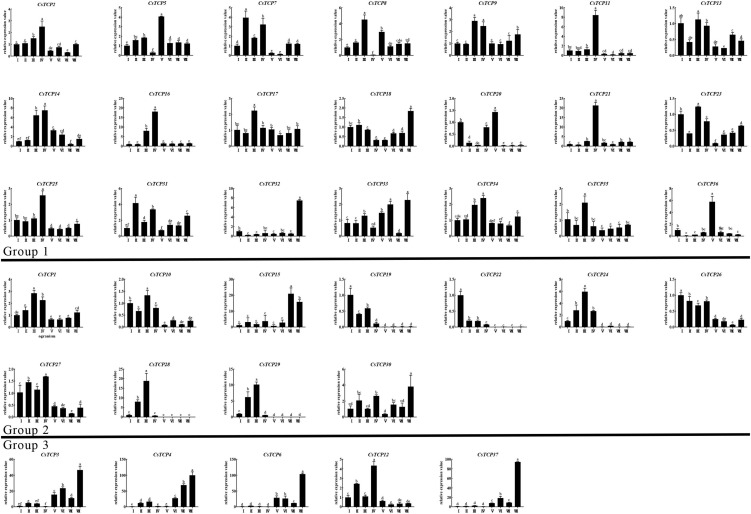
*CsTCP* genes expression patterns in eight tissues.

The expression patterns of *CsTCP* gene family in different tissues did not show significant differences among three groups (Figure 7). In root (IV), *CsTCP1/CsTCP2/CsTCP7/CsTCP9/CsTCP11/CsTCP12/CsTCP14/CsTC P16/CsTCP21/CsTCP25/CsTCP27/CsTCP30/CsTCP31/CsTCP34* were highly expressed. The *CsTCP* gene family is expressed in leaves at different developmental stages. The expression levels of *CsTCP19/CsTCP20/CsTCP22/CsTCP26/CsTCP32/CsTCP36* decreased gradually during leaf maturation, but the expression of *CsTCP1/CsTCP2/CsTCP4/CsTCP5/CsTCP8/CsTCP9/CsTCP14/CsTCP16/CsTCP17/CsTCP21/CsTCP24/CsTCP28/CsTCP29/CsT CP31/CsTCP33/CsTCP34/CsTCP35* increased gradually.

The expression level of *CsTCP* genes in stems showed different characteristics in tender phloem (V), old phloem (VI), tender xylem (VII), and old xylem (VIII) (Figure 7). In phloem, the expression level of *CsTCP5/CsTCP8/CsTCP14/CsTCP20/CsTCP33/CsTCP36* was higher; the expression level of *CsTCP14* was high in old phloem (VI), and the rest was high in tender phloem. In xylem, the expression levels of *CsTCP7/CsTCP9/CsTCP13/CsTCP18/CsTCP23/CsTCP25/CsTCP31/CsT CP32/CsTCP33/CsTCP15/CsTCP3/CsTCP4/CsTCP6/CsTCP30/CsTCP37* were high; among them, *CsTCP15* is highly expressed in tender xylem (VII), and the rest is highly expressed in old xylem. Some *CsTCP* genes have special expression patterns and only show high-level expression in a single plant organ, such as *CsTCP11/CsTCP21/CsTCP22/CsTCP28/CsTCP29/CsTCP32/CsT CP36/CsTCP15/CsTCP3/CsTCP4/CsTCP6/CsTCP37*. Some genes of group 2, such as *CsTCP15/CsTCP19/CsTCP10*, and most genes of group 1, *CsTCP9/CsTCP11/CsTCP13/CsT CP16/CsTCP17/CsTCP5/CsTCP8/CsTCP34/CsTCP20/CsTCP18/CsTCP21/CsTCP23*, were downregulated in varying degrees under drought and salt stress (Supplementary Figure 4). In addition, *CsTCP15/CsTCP19/CsTCP22* of group 2 and *CsTCP9/CsTCP11/CsTCP16/CsTCP17/CsTCP23* of group 1 were upregulated under cold stress (Supplementary Figure 4). These stress responsive genes were also induced by MeJA, and the expression level had no significant correlation with the treatment time (Supplementary Figure 4).

## Discussion

As an important economic crop in China, tea plant and its products have made significant contributions to Chinese agricultural industry. However, the molecular biological mechanisms of tea plant development have seldom been reported. TCP proteins play an important role in plant morphological evolution and development. *TCP* gene family has been identified in many plant species, such as *Arabidopsis thaliana* (Riechmann et al., 2000; Yao et al., 2007), *Oryza sativa*. L (Xiong et al., 2005), *Lycopersicon esulentum Mill* (Parapunova et al., 2014), and *Gossypium raimondii* (Ma et al., 2016). However, the identification of *TCP* gene family in tea plant is controversial and superficial. In this study, a variety of methods were used to identify the *TCP* gene family of tea plant, and the evolutionary process and functional characteristics of CsTCP proteins were analyzed.

### Identification of *TCP* Gene Family in *Camellia sinensis* var. *Sinensis* Genome

In this study, 37 CsTCP proteins with TCP domain were identified, and three new CsTCP proteins CsTCP32, CsTCP33, and CsTCP34 were characterized compared to previous research, where 34 TCPs were found (Yu et al., 2021). Previous studies used the TCP protein of Arabidopsis and rice as queries for local BLAST searches against the TPIA. This method may lead to the elimination of some CsTCP proteins with TCP-conserved domain but low homology with the TCP protein of Arabidopsis and rice.

The residues of CsTCP proteins between class I and class II were definitely different in the loop, helix I, and helix II regions; however, a conserved tandem of tryptophan (W) and leucine (L) was found in helix II (Figure 1), which further indicates that CsTCP proteins may be functional redundancy. Many studies have shown that there is functional redundancy among TCP proteins in same group, for example, JAW-TCPs AtTCP7/AtTCP8/AtTCP22/AtTCP23 of CIN group (Aguilar-Martínez and Sinha, 2013) and AtTCP14/AtTCP15 of PCF group (Ferrero et al., 2021) in Arabidopsis. Thus, the mutation of single *TCP* gene will not cause plant phenotypic changes, such as *AtTCP4*/*AtTCP10* (Koyama et al., 2017), *BrrTCP2* (Du et al., 2017), and *SlLA* (Shleizer-Burko et al., 2011). Then, we speculate that the similar situation could happen in tea plant. Most TCP proteins have obvious differences in motif composition in tea plant, for example, motifs 2, 3, 5, 6, and 8 (Figure 3B). The special motif composition among different groups supports the functional differentiation of CsTCP protein. We conclude that CsTCP proteins in different groups are supposed to have complementary functions, whereas those in the same class could display the function redundancies, and the phylogenetic distribution of CsTCP proteins in the evolutionary tree among species is also supported this result (Figure 4).

### *TCP* Gene Family in *Camellia sinensis* and Their Evolution

In this study, we found that there was no relationship between the number of *CsTCP* genes and the genome size (Table 2). Moreover, the number of *TCP* genes is increased with the evolution of species from lower to higher, and the *TCP* gene family has been expanded in higher plant (Martin-Trillo and Cubas, 2010). From the phylogenetic tree with six species, we observed that there were 11 well-supported clades in both tea plant and rice or maize genes (Figure 6), suggesting that the most recent common ancestor of eudicots and monocots had at least 11 *TCP*-conserved genes, because there are a few additional clades in only eudicots or monocots (rice and maize) genes, indicating that some *TCP* genes may lost. The number of *TCP* genes in the recent revolved plant species is probably over 11 (Yao et al., 2007). Therefore, the *TCP* gene family has only expanded since the divergence of monocots and eudicots in plant evolution history.

In plant genome, gene duplication and divergence are the essential steps for the gene family expansion and evolution of new function. To evaluate the effect of duplication on the *CsTCP* gene family, we first analyzed the duplicate events in *CsTCP* gene family. The results showed that 95% (35/37) *CsTCP* genes were duplicated from WGD/segmental event, and 30% (11/37) were also duplicated from transposed event (Supplementary Table 2). Transposed genes in tea plant are collinear with the genome ancestral plant species. The transposed genes were distributed in the 11 clades of the phylogenetic tree (Figure 4), suggesting that these genes are relatively conservative in the evolution. Moreover, thirteen syntenic pairs were detected in tea plant. The results demonstrated that WGD/segmental duplication played a vital role in the expansion of the *CsTCP* gene family.

To explore the evolution of *CsTCP* gene family, we analyzed their syntenic pairs in tea plant and between tea plant and grapevine. A total of nineteen *CsTCP* genes have counterparts in syntenic pairs (Figures 5, 6). A total of 11 of them belong to class I and eight belong to class II. The syntenic analysis between tea plant and grapevine showed that these genes located in corresponding syntenic blocks occurred before the divergence of tea plant and grapevine. In addition, previous study showed that, after core eudicot whole-genome triplication (WGT) with *Vitis vinifera*, *C. sinensis* has experienced additional WGD event (Chen et al., 2020b). Tea plant has experienced additional duplication event, resulting in a further increase of *CsTCP* gene numberin two classes, but the process of the event remains to be further studied.

### Expression Profile Analysis of *TCP* Gene in *Camellia sinensis*

The *CsTCP* genes from group 3 are highly expressed in buds and stems (Figure 7 and Supplementary Figure 4), especially in the lignified stems (VII, VIII). The *CsTCP* genes from group 2 are mainly expressed in buds, flowers, and leaves (Figure 7 and Supplementary Figure 4). This is similar to the expression pattern in tomato. The expression levels in different organs vary widely between the tomato *TCP* genes, as well as between different organs for individual *TCP* genes (Parapunova et al., 2014). Most *CsTCP* genes of group 1 are more widely and non-specifically expressed in different tissues, as well as in tomato; the difference is that the *CsTCP* genes are expressed in all tissues, including buds, flowers, fruits, leaves, and stems (Supplementary Figure 4), whereas the *SlTCP* genes are mainly expressed in leaves, flowers, and fruits (Parapunova et al., 2014). It may be caused by the difference of the number of *TCP* genes in species. The *CsTCP* genes expression pattern in tissues are also similar to *ZmTCP* genes in maize that contains a large number of *TCP* genes (Ding et al., 2019).

*CsTCP* genes of group 3 basically did not show any expression difference in response to stress. This is similar to *ZmTCP* genes, and most of *ZmTCP* genes (13/19) of CYC/TB1 (group 3) in maize do not respond to stress induction (Ding et al., 2019). The expression levels of many CsTCP genes (16/32) changed under stress treatment, and these genes (12/16) mainly belong to PCF group. The result was also supported by the study of PCF-*TCP* genes in many species. In rice, most of the PCF group (group 1) genes participate in the stress response, *OsPCF6* and *OsTCP21* expression were largely induced by cold stress, and the downregulation of *OsPCF6* and *OsTCP21* resulted in enhanced tolerance to cold stress (Wang et al., 2014), and OsPCF5/OsPCF8 (Yang et al., 2013) and OsTCP19 (Mukhopadhyay and Tyagi, 2015) play the important roles in the stress response. In other plant species, *TCP* genes involved in abiotic stress response mostly belong to PCF group, such as in *Phyllostachys edulis* (Liu et al., 2020), *Glycine max* (Ling et al., 2020), *Betula platyphylla* (Li et al., 2020; Ren et al., 2021), and so on.

In the evolutionary tree, TCP family of tea plant and Arabidopsis can be divided into 9 clades (Supplementary Figure 5). The *TCP* genes of clades 8 and 9 belong to CIN group, and their expression levels are higher in leaves (Figure 7 and Supplementary Figure 4). CIN-*TCPs* have been found to play an important role in leaf development in Arabidopsis, including leaf primordium initiation (Alvarez et al., 2016), leaf expansion (Nath et al., 2003), leaf margin formation (Palatnik et al., 2003; Ori et al., 2007; Efroni et al., 2008), and leaf meristem differentiation (Navaud et al., 2007). In Arabidopsis, CIN-*TCPs* are divided into two clades, one is *JAW-TCPs* with miRNA319-binding site, which is regulated by miRNA319, and the other is *TCP5-like* clade without miRNA319-binding site. Clade 8 belongs to *JAW-TCPs* and clade 9 belongs to *TCP5-like* clade. Clade 7 contains *AtBRC* genes, such as *BRC1* (*AtTCP18*) and *BRC2* (*AtTCP12*). *CsTCP3/CsTCP4* are specifically highly expressed in stems (Figure 7 and Supplementary Figure 4), and they fell into the same clade with *AtBRC* in the phylogenetic tree, so these two genes may be *CsBRC1-like*. *CsTCP12* is specifically expressed in leaves (Figure 7 and Supplementary Figure 4) and may be the *CsBRC2-like*. In Arabidopsis, JAW-TCPs and TCP5-like are used as the enhancers for axillary branch growth, and *Branched* genes (*AtTCP12* and *AtTCP18*) are used as the inhibitors to participate in plant branch development (Aguilar-MARTíNEZ et al., 2007; van Es et al., 2019). The function of *TCP* genes related to tea plant leafing, branching, and stress response in tea plant needs to be further studied.

In addition, we also found two interesting clades, clade 6 and clade 2 (Supplementary Figure 5). The number of *CsTCP* genes in most clades is large, but clade 6 contains only one *CsTCP* gene (*CsTCP11*) and three *AtTCP* genes (*AtTCP8/AtTCP22/AtTCP23*). A total of three *AtTCP* genes are involved in regulating leaf development, and there are redundancy functions among them (Danisman et al., 2013). *CsTCP11* is highly expressed in lateral buds, fruits, and roots and induced by stress (Supplementary Figure 4), which seems to have different functions with the *AtTCP* genes in clade 6. Clade 2 contains only one *AtTCP* gene (*AtTCP11*) and five *CsTCP* genes (*CsTCP32/CsTCP33/CsTCP34/CsTCP35/CsTCP36*). The expression levels of these *CsTCP* genes in clade 2 are low under different tissues and stress treatments; however, their expression could be induced under MeJA treatment (Figure 7 and Supplementary Figure 4). The expression of *CsTCP33* could not be detected under these treatments. *CsTCP32/CsTCP33/CsTCP34* also did not exist in the other two tea varieties HD and TGY (Supplementary Table 1). It shows that these three genes either play a role in variety specificity or are non-functional genes.

## Data Availability Statement

The datasets presented in this study can be found in online repositories. The names of the repository/repositories and accession number(s) can be found below: https://doi.org/10.6084/m9.figshare.19291454.v1.

## Author Contributions

WF, YM, XZ, and XS designed the experiment. YM, XS, DZ, HQ, YW, and ZH performed the experiment. YM, XS, and LZ performed the search strategy and analyzed the data. YM and XS wrote the manuscript. ZZ, XZ, and WF paid for part of the study and provided revised suggestions. All authors read and approved the final manuscript.

## Conflict of Interest

The authors declare that the research was conducted in the absence of any commercial or financial relationships that could be construed as a potential conflict of interest.

## Publisher’s Note

All claims expressed in this article are solely those of the authors and do not necessarily represent those of their affiliated organizations, or those of the publisher, the editors and the reviewers. Any product that may be evaluated in this article, or claim that may be made by its manufacturer, is not guaranteed or endorsed by the publisher.

## Footnotes

1
https://www.Arabidopsis.org/

2
http://pfam.xfam.org/

3
http://tpia.teaplant.org/index.html

4
http://smart.embl.de/

5
http://planttfdb.cbi.pku.edu.cn/

6
http://rice.plantbiology.msu.edu/index.shtml

7
http://bioinfo.sibs.ac.cn/Am/index.php

8
https://web.expasy.org/protparam/

9
http://linux1.softberry.com/berry.phtml

10
https://meme-suite.org/meme/tools/meme

11
http://bioinformatics.psb.ugent.be/webtools/plantcare/html/

12 https://github.com/tanghaibao/jcvi/wiki/MCscan-(Python-version)
