# Supplementary material for: Genome-Wide Analysis of the TCP Gene Family and Their Expression Pattern Analysis in Tea Plant (Camellia sinensis)
Source: Front Plant Sci. 2022 Jul 1;13:840350. doi: 10.3389/fpls.2022.840350 (PMC9284231; doi:10.3389/fpls.2022.840350)
Supplement: Supplementary file 1 [file Data_Sheet_1.docx]

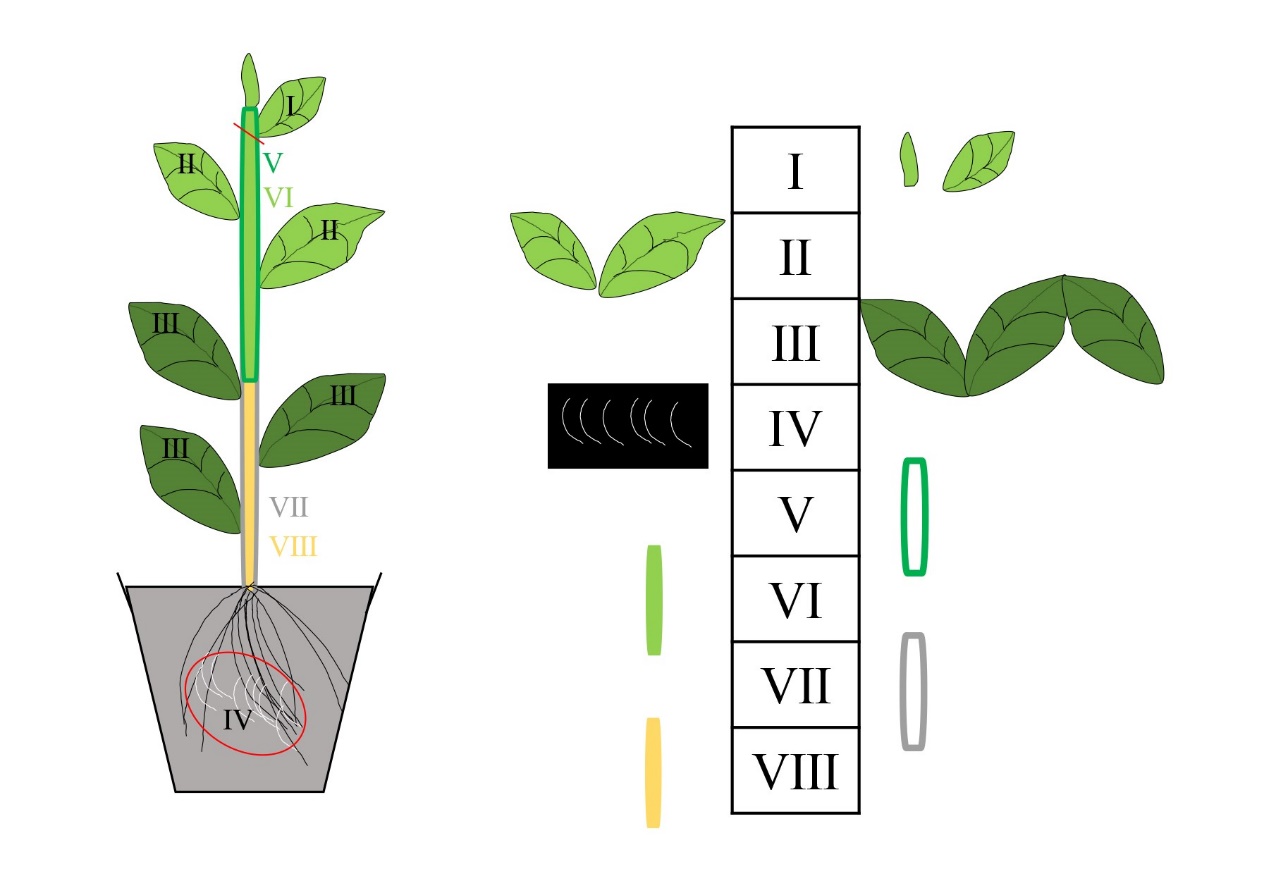


Figure S1 Sampling site of tea seedling


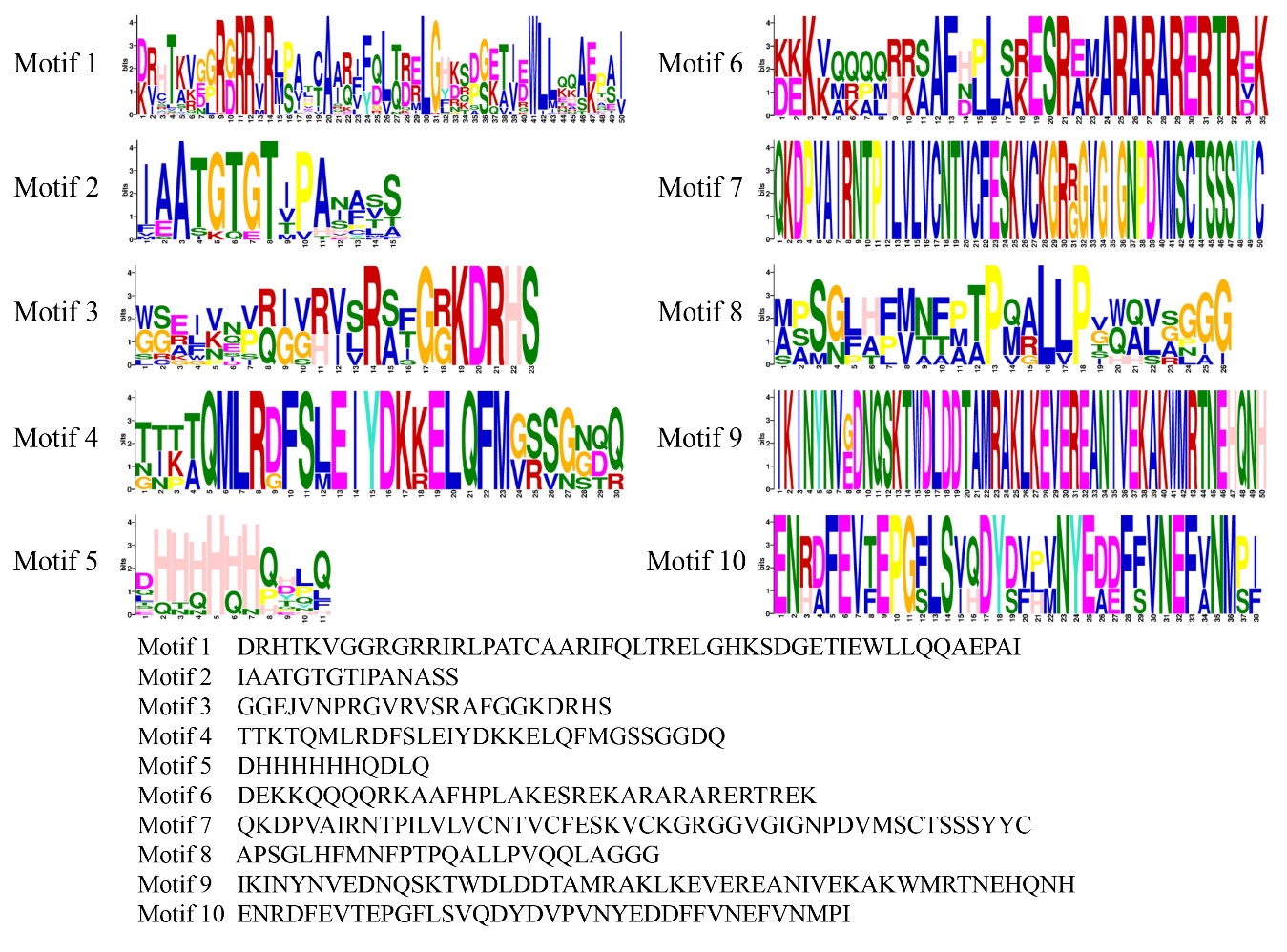


Figure S2 the motif of CsTCP proteins


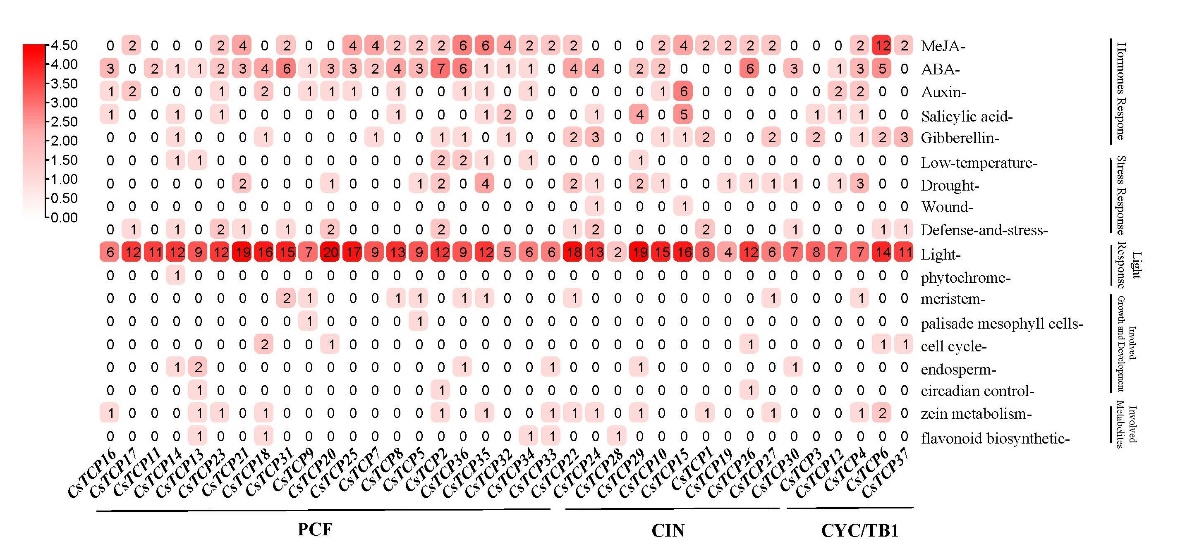


Figure S3 Number of every cis-regulatory element in the promoter region of *CsTCPs*.


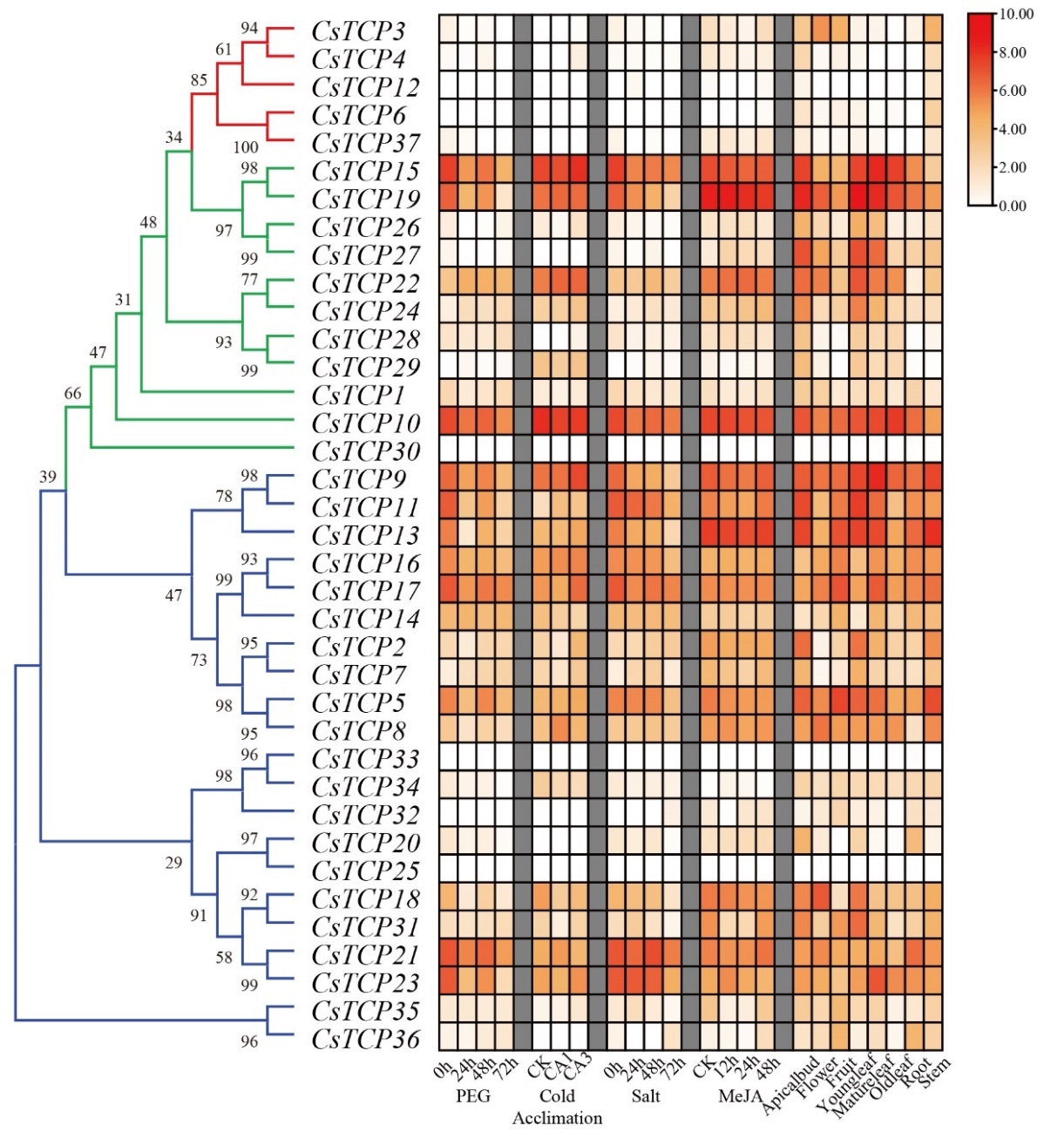


Figure S4 Expression pattern of CsTCP genes in TPIA

PEG：drought stress; Cold Acclimation: cold stress^[80]^; Salt: Salt stress; MeJA: MeJA treatment;


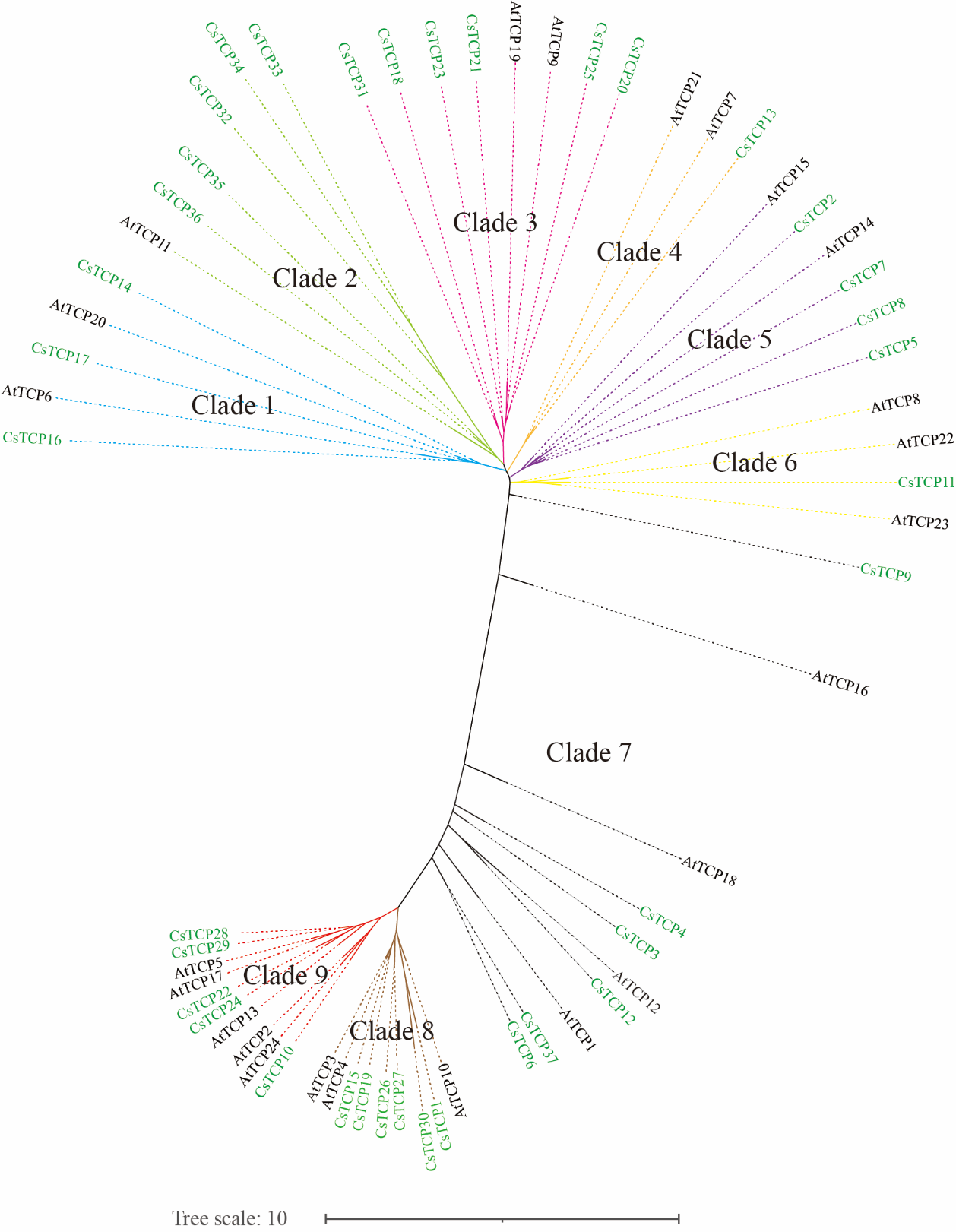


Figure S5 Phylogenetic tree constructed by CsTCPs and AtTCPs

| Table S1 Identification of TCP gene family in different tea varieties | | | |
| --- | --- | --- | --- |
| **HD Gene ID** | **TGY Gene ID** | **TCP gene name** | **SCZ Gene ID** |
| GWHPAZTZ025790 | GWHPASIV027071 | CsTCP1 | TEA018731.1 |
| GWHPAZTZ042949 | GWHPASIV045161 | CsTCP2 | TEA014952.1 |
| GWHPAZTZ043244 | GWHPASIV045469 | CsTCP3 | TEA011977.1 |
| GWHPAZTZ005264 | GWHPASIV005530 | CsTCP4 | TEA021545.1 |
| GWHPAZTZ035117 | GWHPASIV037046 | CsTCP5 | TEA019201.1 |
| GWHPAZTZ005421 | GWHPASIV005713 | CsTCP7 | TEA028581.1 |
| GWHPAZTZ017677 | GWHPASIV018633 | CsTCP8 | TEA013742.1 |
| GWHPAZTZ008088 | GWHPASIV008559 | CsTCP9 | TEA003322.1 |
|  | GWHPASIV040729 |  |  |
| GWHPAZTZ014082 | GWHPASIV026309 | CsTCP10 | TEA033771.1 |
| GWHPAZTZ025043 | GWHPASIV015011 |  |  |
| GWHPAZTZ014076 |  |  |  |
| GWHPAZTZ014078 |  |  |  |
| GWHPAZTZ012199 | GWHPASIV013181 | CsTCP11 | TEA003956.1 |
| GWHPAZTZ017933 | GWHPASIV018906 | CsTCP12 | TEA027566.1 |
| GWHPAZTZ034288 | GWHPASIV036121 | CsTCP13 | TEA012894.1 |
|  | GWHPASIV040948 |  |  |
| GWHPAZTZ029557 | GWHPASIV031010 | CsTCP14 | TEA013055.1 |
| GWHPAZTZ014804 |  | CsTCP15 | TEA021348.1 |
| GWHPAZTZ003941 | GWHPASIV004259 | CsTCP16 | TEA032594.1 |
| GWHPAZTZ003796 |  | CsTCP17 | TEA027172.1 |
| GWHPAZTZ019320 | GWHPASIV020294 | CsTCP18 | TEA015531.1 |
| GWHPAZTZ021364 | GWHPASIV022310 | CsTCP19 | TEA015978.1 |
| GWHPAZTZ022374 | GWHPASIV024587 | CsTCP20 | TEA024520.1 |
|  | GWHPASIV024418 |  |  |
| GWHPAZTZ036200 | GWHPASIV038069 | CsTCP21 | TEA014746.1 |
| GWHPAZTZ004274 | GWHPASIV004565 | CsTCP22 | TEA015233.1 |
| GWHPAZTZ040435 | GWHPASIV042326 | CsTCP23 | TEA007156.1 |
| GWHPAZTZ003342 | GWHPASIV003506 | CsTCP24 | TEA014573.1 |
| GWHPAZTZ023551 | GWHPASIV024419 | CsTCP25 | TEA000693.1 |
| GWHPAZTZ022047 | GWHPASIV023000 | CsTCP26 | TEA018080.1 |
| GWHPAZTZ005319 | GWHPASIV005614 | CsTCP28 | TEA005508.1 |
|  | GWHPASIV045338 | CsTCP29 | TEA030615.1 |
| GWHPAZTZ009988 | GWHPASIV010803 | CsTCP31 | TEA017971.1 |
| GWHPAZTZ013489 | GWHPASIV014408 | CsTCP35 | TEA025851.1 |
| GWHPAZTZ030906 | GWHPASIV032349 | CsTCP36 | TEA033591.1 |
| GWHPAZTZ043105 |  | CsTCP13-like |  |
| GWHPAZTZ025391 |  | CsTCP13-like |  |
| GWHPAZTZ038394 |  | CsTCP13-like |  |
| GWHPAZTZ033171 |  | CsTCP13-like |  |

Note: HD is Camellia sinensis var. sinensis cv. Huangdan; TGY is Camellia sinensis var. sinensis cv. Tieguanyin; SCZ is Camellia sinensis var. sinensis cv. Shuachazao

Table S2 *CsTCP* gene duplication events

| Transposed duplication | | | |
| --- | --- | --- | --- |
| Transposed | Location | Parental | Location |
| CsTCP9 | chr6:181399954 | CsTCP7 | chr8:76436042 |
| CsTCP10 | chr5:23784007 | CsTCP22 | chr8:14892406 |
| CsTCP11 | chr1:125449837 | CsTCP5 | chr14:99259894 |
| CsTCP13 | chr14:2254036 | CsTCP7 | chr8:76436042 |
| CsTCP25 | chr9:12617464 | CsTCP20 | chr9:22204137 |
| CsTCP28 | chr3:255390857 | CsTCP29 | chr8:72227362 |
| WGD | | | |
| Duplicate 1 | Location | Duplicate 2 | Location |
| CsTCP1 | chr5:37542053 | CsTCP19 | chr9:179191838 |
| CsTCP1 | chr5:37542053 | CsTCP27 | chr9:145547453 |
| CsTCP1 | chr5:37542053 | CsTCP30 | chr5:158829592 |
| CsTCP2 | chr15:33757126 | CsTCP8 | chr2:203351583 |
| CsTCP2 | chr15:33757126 | CsTCP7 | chr8:76436042 |
| CsTCP3 | chr15:12688408 | CsTCP12 | chr2:192256395 |
| CsTCP3 | chr15:12688408 | CsTCP6 | chr3:239610103 |
| CsTCP3 | chr15:12688408 | CsTCP4 | chr8:68958208 |
| CsTCP5 | chr14:99259894 | CsTCP2 | chr15:33757126 |
| CsTCP5 | chr14:99259894 | CsTCP8 | chr2:203351583 |
| CsTCP5 | chr14:99259894 | CsTCP7 | chr8:76436042 |
| CsTCP6 | chr3:239610103 | CsTCP4 | chr8:68958208 |
| CsTCP8 | chr2:203351583 | CsTCP7 | chr8:76436042 |
| CsTCP12 | chr2:192256395 | CsTCP4 | chr8:68958208 |
| CsTCP12 | chr2:192256395 | CsTCP6 | chr3:239610103 |
| CsTCP14 | chr12:181008851 | CsTCP16 | chr8:1300774 |
| CsTCP14 | chr12:181008851 | CsTCP17 | chr3:261939741 |
| CsTCP15 | chr4:31849470 | CsTCP1 | chr5:37542053 |
| CsTCP15 | chr4:31849470 | CsTCP19 | chr9:179191838 |
| CsTCP15 | chr4:31849470 | CsTCP27 | chr9:145547453 |
| CsTCP17 | chr3:261939741 | CsTCP16 | chr8:1300774 |
| CsTCP18 | chr2:113112489 | CsTCP31 | chr6:24205113 |
| CsTCP21 | chr14:164452217 | CsTCP20 | chr9:22204137 |
| CsTCP21 | chr14:164452217 | CsTCP31 | chr6:24205113 |
| CsTCP22 | chr8:14892406 | CsTCP29 | chr8:72227362 |
| CsTCP23 | chr13:65540504 | CsTCP20 | chr9:22204137 |
| CsTCP23 | chr13:65540504 | CsTCP21 | chr14:164452217 |
| CsTCP23 | chr13:65540504 | CsTCP31 | chr6:24205113 |
| CsTCP24 | chr3:243796584 | CsTCP22 | chr8:14892406 |
| CsTCP24 | chr3:243796584 | CsTCP29 | chr8:72227362 |
| CsTCP27 | chr9:145547453 | CsTCP19 | chr9:179191838 |
| CsTCP28 | chr3:255390857 | CsTCP29 | chr8:72227362 |
| CsTCP32 | chr11:63307633 | CsTCP36 | chr12:63933857 |
| CsTCP33 | chr11:132048323 | CsTCP34 | chr2:60022992 |
| CsTCP35 | chr1:212317077 | CsTCP36 | chr12:63933857 |

| Table S3 Primes of *CsTCP* genes for qPCR | | |
| --- | --- | --- |
| Gene | Forward prime | Reverse prime |
| *CsTCP1* | AGACGGCCAAGCTACGAC | CACCGCCAAATCAACTCC |
| *CsTCP2* | TGGCGGTGGTGGAAGTAGTG | GGTGGCTAGTTGTGTCTTGTCG |
| *CsTCP3* | AGAGGACAAGAGACAAGCGAAG | AACATCAGCAAGCACATCAAGG |
| *CsTCP4* | AATGGCAACAACACCAACCC | GTACCCTCAACGGCTGTCAT |
| *CsTCP5* | CATCCGCATGCCAGCAACTT | TGCAGACATGGTAGAGCCGG |
| *CsTCP6* | TCCAGTTCCACCATAGCA | GCCTCCATTGTCATCAGAA |
| *CsTCP7* | CGCACCGTCTCGCTTCAACAG | CCCGTTTCCGCTCCATACTCTCC |
| *CsTCP8* | AAGTCCGATGGAGAGACCAT | TGATGCCGTGGAGAGATTCG |
| *CsTCP9* | TAGCCATCGCCACCAGATCC | TTCCGTCGACTTTGGTGTGG |
| *CsTCP10* | GTCACCACCGCTATTCAGTT | CCCTTGTTCAGTACCAGCAC |
| *CsTCP 11* | CTCCAAAGACCGCCACACTAAG | GCCTGTTGAAGAAGCCACTCG |
| *CsTCP 12* | GCACTTCCCTTCACCTTTTCTTG | CCGTCATCAGTTCTCTTCTTCCTC |
| *CsTCP 13* | CCGTCAGAAACTCCTCCAAT | AACTCCATACCTGGCCGAAG |
| *CsTCP 14* | TCGGCTCTTGCAGCTACAGG | CATGGAAGCCCAATTTAGAC |
| *CsTCP 15* | CACCGCCATTCAGTTCTAC | GCTCGTCAGTTCTTGTTCG |
| *CsTCP 16* | AACCATTCAGTGGCTCTTAC | GCCCTACCACCCAATTCATC |
| *CsTCP 17* | TTCGGATGCCAGCACTCTGC | GATGCAATCCCACCGATATG |
| *CsTCP18* | CCAACTCACCCGAGAACTCG | GCGTTTCTTCTTTGTGGTGG |
| *CsTCP19* | TCAGCAGCAGCATCAGAC | CCATTCCACGCAACCATTC |
| *CsTCP20* | GAATCAGCCTCAGATATGGT | GGTGGTGGTTGAACTTGA |
| *CsTCP21* | CAAAGGACCGCCATACGAAG | TGGCTTGCTCAGCGTGTTCT |
| *CsTCP22* | AGGCTTGGGCTTAATCAGCC | AGGCCTTCTTTGTCTCCTAG |
| *CsTCP23* | CCTATCTCGTCGTTCGTATC | CACCGTATCCACCGTCTC |
| *CsTCP24* | GCTGAATGAAGCTAAGCATG | TAGTCCCAATGGAGTGTCCC |
| *CsTCP25* | ATGACGCCGCAACGACTG | TGGAGCCATTATTGACACCTT |
| *CsTCP26* | GCGGTGGAAATGGAGAGAATAG | TGGTGGAGATTGAAGATTGTTGG |
| *CsTCP27* | GAGATTGGTGTCGTGGAATGG | GGTTGTGGTGGCTGAAATGG |
| *CsTCP28* | GCGAAGTGGATGAGAACA | GCTAACGAAGATGGCAATG |
| *CsTCP29* | TGCCAATGCCATACAACTA | TGAAGCGACGACATAAGG |
| *CsTCP30* | TCTCTTCCTCTCCCTCCTCTC | TGCCATAGCCAAGCCTGTC |
| *CsTCP31* | GATCTTCCAACTAACCCGAG | AGGGTTCCGCCGACAGACAT |
| *CsTCP32* | GACTATTCTTTCTACCCCAT | GTGTTGTAGAGGATTTTGCT |
| *CsTCP33* | CACCACCACCTCACAACCAAC | GAATGCGGATACGACGACCTC |
| *CsTCP34* | CGTAATTCAGGTCTATTCGTTGG | TTCAAACAAAGCCCTAACATCC |
| *CsTCP35* | AAAGACCGCCACACGAAAGT | ATGTCCACCACCAGAAACCC |
| *CsTCP36* | CCACCGCAACTTCTCTTC | GCCGTTCACCTTCGTATG |
| *CsTCP37* | GCACAAGAAGGCTTAATGAT | TTCACAACAGAATCCTCAGT |
| Cs*β*-actin | GCCATCTTTGATTGGAATGG | GGTGCCACAACCTTGATCTT |
